# South Sudan: a young country’s fight against COVID-19

**DOI:** 10.11604/pamj.supp.2020.37.1.27327

**Published:** 2020-12-17

**Authors:** Daniel Garang Aluk Dinyo, Attaullah Ahmadi, Melody Okereke, Mohammad Yasir Essar, Don Eliseo Lucero-Prisno

**Affiliations:** 1 Médecins Sans Frontières-France South Sudan Mission, Juba, South Sudan,; 2 Medical Research Center, Kateb University, Kabul, Afghanistan,; 3 Faculty of Pharmaceutical Sciences, University of Ilorin, Kwara State, Nigeria,; 4 Kabul University of Medical Sciences, Kabul, Afghanistan,; 5 Department of Global Health and Development, London School of Hygiene and Tropical Medicine, London, United Kingdom,; 6 Faculty of Management and Development Studies, University of the Philippines (Open University), Los Baños, Laguna, Philippines

**Keywords:** South Sudan, COVID-19, responses, challenges, conflict, health system

## Abstract

COVID-19 is a highly infectious disease that has started to creep into African countries including South Sudan. Following confirmation of the first few cases, the government enacted preventive measures to curb community transmission. However, daunting challenges deter these precautionary measures. Just after two years the country took its independence from Sudan, civil conflicts sparked and continue to overburden and undermine the fragile healthcare system. The conflicts have caused disruption of health services, destruction of health facilities, death and migration of health workers, displacements of a huge number of people. This scenario continues while the country is grappling with the pandemic. Other concerning issues include: insufficient COVID-19 testing capacity, limited medical and personal protective equipment and an inadequate number of health workers which leave the country ill-equipped in the battle against the pandemic. Non-compliance of COVID-19 prevention protocols by the general public due to high rate of poverty and social stigma contribute to the spread of the virus. The current situation in South Sudan make evident that there is a need for an immediate ceasefire by the warring sides so the available health services including COVID-19 efforts, are not disrupted to ensure the safety of all. The government needs to further build the capacity of its health sector with the cooperation of its international health allies to be able to provide its citizens with the health services they need.

## Commentary

Novel coronavirus disease (COVID-19) is a public health threat of international concern [1], afflicting many countries across the world including African countries. South Sudan confirmed its first case of COVID-19 on 5^th^ April 2020. It was a 29 years old female returnee from the Netherlands, making it the 44^th^ African country infected with the virus. Following the confirmation of the first few cases, the concerns of South Sudan´s National Ministry of Health and its health partners including the World Health Organization (WHO) were raised, thus, some preventive measures were devised to curb the spread of the virus. These precautionary measures included the ban on public gatherings, closure of all academic institutions, suspension of local and international flights and borders [2], imposition of nationwide curfew, closure of religious institutions and decongestion of some prisons [3]. These preventive measures were timely and similar to those implemented by some developed countries, but not the sole determinants of the course of the pandemic. Thus, in this article, we aim to provide a critical commentary on a series of challenges that couple to form the underlying factors in undermining the country´s healthcare system and, thus, deterring the containment efforts.

Conflict severely disrupts health interventions, deprive people of health services and cripple health systems [4]. Just two years after South Sudan took her independence from Sudan, the conflicts sparked and since then continues to be a formidable threat for the country´s healthcare system and damages it on a large scale. A huge number of health facilities have been targeted and destructed to ashes by various warring sides [5]. Many professionals and trained health staff left the country due to the violence as they do not opt to work in insecure areas [5]. Conflicts affect health systems by hampering disease management strategies and efforts and ultimately result in forced displacement and migration. It also creates the burden of infectious diseases and disrupts public health responses. International and national Non-Governmental Organizations (NGOs) with the cooperation of humanitarian agencies have tried for years to provide health services for underprivileged communities, however, their efforts have been threatened by insecurity and the lives of some of their workers have also been claimed. A spate of conflicts has invariably resulted in geographical displacement of affected populations, loss of lives, and properties [2]. The internally displaced people are more vulnerable to the infective diseases including COVID-19. Amidst the pandemic, around one and half million people have limited access to basic health services and inhabit in conditions in which social distancing is not possible [3]. The geographical displacements may make social distancing very difficult to adhere to as populations may ignore social distancing protocols while moving in clusters and congested public transport systems in quest for better living conditions and comfortable settlements. People have been longing for peace and stability so, among other benefits associated with it, they would also receive the essential health services without any disturbance. However, most peace negotiations and accords were merely on the papers and hence a failure.

The preparedness of the youngest country of the world in responding efficiently to the pandemic is waned due to its weak healthcare system. Most of the health facilities are ill- equipped [6,7] and cannot meet the demands of the people, exemplified by the presence of only one permanent hospital ward with less than 100 beds, to treat infectious diseases of the 11 million population [3]. Over half of the government´s healthcare facilities are malfunctioned and understaffed. The country is facing shortages of intensive care units (ICU) and mechanical ventilators [8] and a handful of oxygen concentrators exist for the management of severe cases. WHO´s Global Health Workforce Alliance states that 22.8 skilled health workers per 10,000 population are needed to perform all the essential health interventions. However, there are less than 10 disproportionately distributed health workers per 10,000 people in the country [9]. High level of illiteracy and unawareness are other barriers against the containment efforts.

As it was envisaged that the pandemic in South Sudan would reach its peak in July and August [8], the disease transmission reached significantly high proportions. As of 7^th^ December 2020, the country reported 3,166 confirmed cases and 61 deaths [10], most of which have been reported after relaxation of the restrictions on 7^th^ May 2020 [3]. The current indicators show that the virus has been spreading in the country particularly in vulnerable places like six crowded United Nations´ camps for displaced people in South Sudan, three of which lack screening at their gates [3]. The pandemic has crippled the young nation´s health system, economy, and overall livelihood [2]. Poverty and hunger have become the order of the day and the people are merely struggling to survive and many found themselves forced to violate the restrictions in quest for food and survival [8] due to lack of the government´s assistance [3]. Insufficient testing capacity coupled with social stigma [3,7] and non-compliance of COVID-19 prevention protocols such as self-isolation by the people has made the accurate documentation and containment of the disease prevalence difficult.

The present situation of the COVID-19 pandemic in South Sudan validates the prediction made by the United Nations that the pandemic will lead to an overwhelming increase in deaths [8] due to multiple factors tied to a lack of vaccination services of preventable diseases, decrease in maternal health services, and disruption of routine management of common diseases such as malaria, pneumonia, diarrhea [7,8] and malnutrition [2]. People die from preventable diseases such as measles, cholera, and malaria at the hospitals due to lack of health services. Another concerning issue of public health worth highlighting is the condition of healthcare providers in South Sudan in light of the COVID-19 pandemic. Due to the shortage of personal protective equipment (PPE), 86 health workers in the country were infected as of 23^th^ June 2020 [7]. Also, the salaries of health workers have been left unpaid ever since the inception of their services in the battle against the pandemic [7]. A possible implication is that this will have a corresponding impact on the quality of healthcare delivery due to a generally low level of motivation and compromised safety.

## Conclusion

The current situation in South Sudan requires the warring sides to reach an agreement on an immediate ceasefire so the available health services including COVID-19 efforts are not disrupted to ensure the safety of all. The country is ill-equipped in providing its citizens with health services. Thus, the government needs to further build the capacity of its health sector with the cooperation of its international health allies.
